# Conjecturing about Small-Molecule Agonists and Antagonists of α4β1 Integrin: From Mechanistic Insight to Potential Therapeutic Applications

**DOI:** 10.3390/biomedicines12020316

**Published:** 2024-01-30

**Authors:** Tingting He, Daria Giacomini, Alessandra Tolomelli, Monica Baiula, Luca Gentilucci

**Affiliations:** 1 Department of Chemistry “G. Ciamician”, University of Bologna, Via Gobetti 83, Ue4, 40129 Bologna, Italy; tingting.he2@unibo.it (T.H.); daria.giacomini@unibo.it (D.G.); alessandra.tolomelli@unibo.it (A.T.); 2 Department of Pharmacology and Biotechnology (FABIT), University of Bologna, Via Irnerio 48, 40126 Bologna, Italy; monica.baiula@unibo.it; 3 Health Sciences & Technologies (HST) CIRI, University of Bologna, Via Tolara di Sopra 41/E, 40064 Ozzano Emilia, Italy

**Keywords:** α4β1 integrin, inflammation, crystal structure, molecular docking, agonist

## Abstract

Integrins are heterodimeric cell-surface receptors that regulate cell–cell adhesion and cellular functions through bidirectional signaling. On the other hand, anomalous trafficking of integrins is also implicated in severe pathologies as cancer, thrombosis, inflammation, allergies, and multiple sclerosis. For this reason, they are attractive candidates as drug targets. However, despite promising preclinical data, several anti-integrin drugs failed in late-stage clinical trials for chronic indications, with paradoxical side effects. One possible reason is that, at low concentration, ligands proposed as antagonists may also act as partial agonists. Hence, the comprehension of the specific structural features for ligands’ agonism or antagonism is currently of the utmost interest. For α4β1 integrin, the situation is particularly obscure because neither the crystallographic nor the cryo-EM structures are known. In addition, very few potent and selective agonists are available for investigating the mechanism at the basis of the receptor activation. In this account, we discuss the physiological role of α4β1 integrin and the related pathologies, and review the few agonists. Finally, we speculate on plausible models to explain agonism vs. antagonism by comparison with RGD-binding integrins and by analysis of computational simulations performed with homology or hybrid receptor structures.

## 1. Introduction

**Figure 1:**
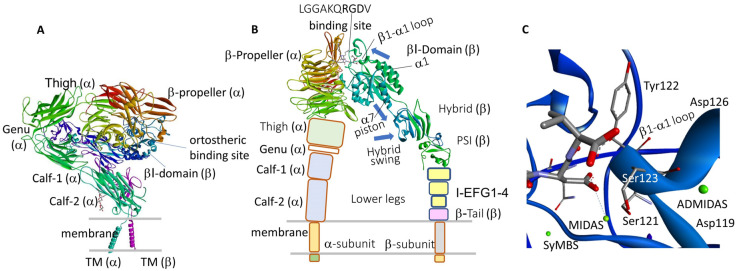
(**A**) Closed structure of full αIIbβ3 integrin (8T2V) [8]; densities are not visible for the αIIb and β3 short cytoplasmic tails. (**B**) Open-extended conformation of αIIbβ3 ectodomain (four domains) in the presence of LGGAKQRGDV (2VDR) [9]; blue arrows indicate the directions of the main displacements underwent by elements of the βI- and hybrid domains during conformational reorganization; PSI, plexin-semaphorin-integrin. No density is detected for thigh, leg, and TM domains. (**C**) Inset of carboxylate binding site in the β-subunit and the metal ion-dependent adhesion sites (green spheres) (2VDR).

Integrins represent the most important family of transmembrane receptors that “integrate” extracellular with intracellular environments via transmission of signals. These receptors are heterodimers composed of non-covalently bound α and β subunits, and each subunit comprises functionally specialized domains (Figure 1). In humans, the combination of 18 α- and 8 β-subunits generates 24 distinct αβ heterodimers. Integrins can be grouped in four classes, i.e., collagen-binding, Arg-Gly-Asp (RGD)-binding, laminin-binding, and leukocyte integrins [1,2].

The natural ligands of these receptors are large proteins of the extracellular matrix (ECM) with relatively low binding affinity, such as fibronectin (FN), vitronectin (VN), laminin, collagens, or cell adhesion molecules (CAM) expressed on endothelial cells, such as vascular cell adhesion protein 1 (VCAM-1), a member of the immunoglobulin superfamily expressed on endothelial cells that line blood vessels [3]. Most integrins interact with multiple ligands, and conversely, a single integrin ligand, e.g., a protein of the ECM or a CAM, can interact with multiple integrins.

Notably, integrins distinguish their endogenous ligands by coming into contact with very short peptide sequences. The Arg-Gly-Asp (RGD) tripeptide is a highly conserved recognition motif found in diverse ECM proteins, for instance in FN or VN, which binds to several integrins, i.e., αvβ3, αvβ1, αvβ5, αvβ6, αvβ8, α5β1, and α8β1, expressed on cancer cells. Similarly, the αIIbβ3 integrin expressed on platelets binds to the native ligand fibrinogen by the RGD motif [4].

Integrins are not constitutively active and thus cannot always bind to ligands. Instead, the binding of integrins to ligands is controlled and requires the binding of cytoplasmic proteins such as talin and kindlin to the intracellular domains of the integrins (inside-out signaling). These cytoplasmic interactions induce large conformational modifications to the extracellular domain, from bent-inactive to extended-active (Figure 1), passing through one or more intermediate states, which in turn permit ligand binding. At least three major conformers have been observed: bent-closed, open-closed, and open-extended. The binding of ligands to integrin by the extracellular side triggers signaling that directs functions such as cell spreading, motility, survival, proliferation, etc. (outside-in signaling). Through their intracellular domains, integrins link indirectly to actin but also to many other cytoskeletal and proteins and kinases, forming large associations called focal adhesions [5].

Besides the normal physiological functions, excessive integrin can lead to various pathological conditions, encompassing the onset and advancement of cancer, coronary diseases, inflammatory and autoimmune pathologies, etc. Consequently, there are high hopes for antibodies or small molecules that can inhibit integrins as potential drugs. In the last twenty years, more than 130 clinical trials of integrin-targeting molecules have been conducted [6,7].

In spite of great effort, up to now, only a few integrin-based drugs have entered the market. Effective treatments have successfully targeted integrins αIIbβ3, α4β7/α4β1, and αLβ2 for cardiovascular diseases, inflammatory bowel disease/multiple sclerosis, and dry eye disease, respectively.

As a matter of fact, despite promising preclinical results, several integrin-targeting drugs have failed in clinical tests [10]. Inhibitors of αvβ3 and αvβ5 integrin have entered clinical trials as antiangiogenic agents. However, there is evidence that these inhibitors can stimulate tumor growth and tumor angiogenesis, at low (nanomolar) concentrations. For instance, the RGD cyclopentapeptide cilengitide, originally proposed as an antiangiogenic drug [11], has been shown to promote VEGF-mediated angiogenesis at nanomolar concentration, by altering signaling of αvβ3 integrin and vascular endothelial growth factor receptor-2 [12]. The small-molecule firategrast, dual antagonist of α4β1/α4β7 integrins, entered in a phase II trial for multiple sclerosis [13]. High doses were significantly therapeutically effective in reducing lesions. However, low doses were significantly harmful; hence, it is no longer in pharmaceutical development. Also in the field of antithrombotic therapy, oral antagonists of αIIbβ3 integrin [14,15] or longer-term dosing of parenteral inhibitors [16] ended in failure.

These disappointing outcomes can be partly ascribed to the action as partial agonist and induction of the high-affinity, extended-open integrin conformation. In fact, several integrin antagonists can also act as partial agonists, thereby triggering outside-in signaling and paradoxically resulting in adhesion and unfavorable outcomes in patients [17].

The mechanism of signaling transmission by physiological ligands is much better understood for integrins αIIbβ3 and αvβ3. In contrast, the mechanism at the basis of activation and signaling for integrins α4β1 is particularly unclear. This is mostly due to the fact that, at present, there are no reported crystal nor cryo-EM structures.

Herein, we give an updated survey of the currently available information for this integrin, including a review of the few agonists. In particular, the results of computational investigations performed by using homology or composite models of the receptor are discussed and compared with information available for other integrins.

## 2. Biology and Functions of α4β1 Integrin

**Figure 2:**
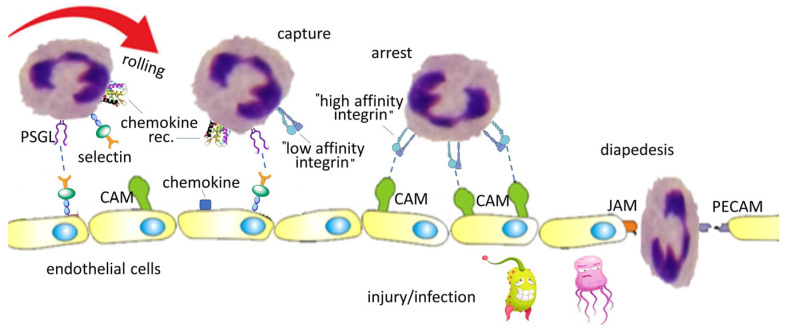
Simplified representation of leukocyte adhesion cascade involved in immune cell infiltration. The process consists of leukocyte rolling, capture, arrest (firm adhesion), and transmigration (diapedesys) from the circulatory system to the infected site. This cascade is mediated by various molecules: selectins and their ligands (PSGL), integrins and the cell adhesion molecules (CAM). Extravasation is driven by junctional adhesion molecules (JAM) and platelet endothelial cell adhesion molecules (PECAM).

In case of tissue injury or infection, the circulating leukocytes are triggered, and migrate to the affected site with the principal aim of eliminating the inflammatory origin and contributing to tissue repair. The movement of leukocytes, for instance neutrophils, from the blood across the endothelium, is a well-organized cascade process, consisting of the following steps (Figure 2): rolling, capture, arrest, and finally transmigration (diapedesys) across the blood vessel wall [18].

Each of these steps is mediated by specific interactions (Figure 2). Initially, selectins (expressed on both leukocytes and endothelial cells) interact with their ligands (e.g., PESGL-1) allowing leukocytes to roll onto the endothelial surface. Chemokines released locally activate integrins via their G-protein coupled receptors (GPCR). Integrins gain affinity for their CAM ligands, and the firm interaction between them brings to leukocyte arrest. Thereafter, leukocytes extravasate to the site of infection, and this movement is mediated by specific adhesion molecules (JAM, PECAM) [19].

The leukocyte subfamily of integrins comprises seven members: four β2 integrins, two α4, and αEβ7 [20]. Their expression varies among the subpopulations of leukocytes. The integrins of the β2 family, such as the αLβ2 (or leukocyte function-associated antigen-1, LFA-1) and αMβ2 (Mac-1), bind to their ligands ICAM-1 (CD54) and ICAM-2 (CD102), respectively [21].

α4β1 Integrin, also known as very late antigen-4 (VLA-4) or CD49d/CD29, is expressed on most leukocytes, and is involved in their homing, trafficking, differentiation, activation, and survival. This integrin recognizes the Leu-Asp-Val-Pro (LDVP) peptide in FN, the Leu-Asp-Thr-Ser (LDTS) sequence in the mucosal addressing cell adhesion molecule-1 (MAdCAM-1), and Ile-Asp-Ser (IDS) in VCAM-1. The α4 subunit is also found together with the β7 subunit in the α4β7 dimer. The natural ligand of α4β7 integrin is MAdCAM-1, whose peptide recognition motif is Leu-Asp-Thr (LDT) [22].

Blocking α4β1 integrin could represent an opportunity for the treatment of diverse disorders [23] because it is involved in the development and sustainment of inflammation, in inflammation-related diseases, and in mobilization or retention of stem cells. This receptor is also implicated in T cell transit through the blood−brain barrier (BBB) in autoimmune encephalitis. In multiple sclerosis (MS), migration of autoreactive T lymphocytes into the central nervous system (CNS) is governed by the interaction between α4β1 integrin and VCAM-1. In allergic conjunctivitis, α4β1 integrin mediates persisting infiltration of neutrophils, eosinophils, and T lymphocytes in the conjunctiva. In asthma and sarcoidosis, this receptor participates in the accumulation of lymphocyte in the lung. α4β1 Integrin is expressed in different varieties of cancer, i.e., multiple myeloma, ovarian cancer, and pancreatic cancer, and recent studies have described its potential role in cancer development and in the formation of metastasis [24].

As for the related α4β7 integrin, interaction of α4β1 integrin with MAdCAM-1 is responsible for T lymphocytes accumulation to the gut; hence, it is a good target for inflammatory bowel diseases, such as ulcerative colitis and Crohn’s disease.

Not surprisingly, this integrin has been targeted for the pharmacological treatment of autoimmune diseases such as MS [25], or inflammatory pathologies. The humanized anti-α4β1 monoclonal antibody (mAb) natalizumab blocks, in the central nervous system (CNS), the interaction of the integrin with adhesion molecules of endothelial cells, and the lymphocyte migration through the BBB, thus effectively preventing formation of new lesions and relapses in MS [26]. However, potential adverse effects of mAb may be severe, including secondary autoimmune diseases or malignancies, and can necessitate treatment discontinuation. Natalizumab was retired from the market in 2005 because its use favoured the insurgence of progressive multifocal leukoencephalopathy (PML) in patients with MS [27]. Subsequently, it was readmitted the following year, with a notice about the increased risk of PML [28].

## 3. α4β1 Integrin Antagonists

As an alternative to antibodies, small-molecule antagonists can be utilized to interfere with integrin–ligand interactions [23]. The small-molecule antagonists of α4β1 integrin reported in the literature can be grouped in a few classes [29].

**Figure 3:**
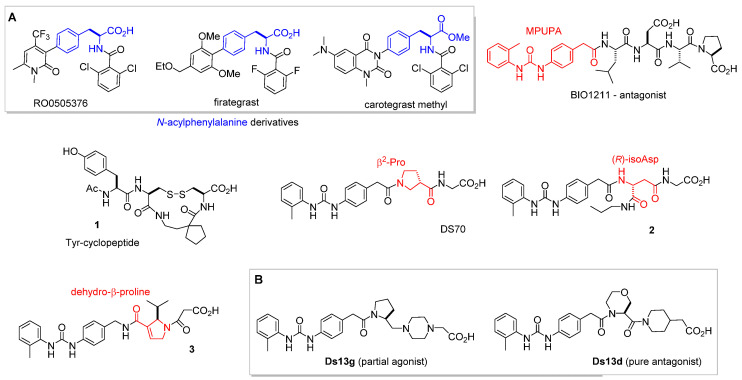
Structures of α4β1 integrin antagonists discussed in this paper: (**A**) *N*-acylphenylalanine derivatives RO0505376, firategrast, carotegrast-methyl; LDV peptide BIO1211; cyclic peptide **1** containing *N*-terminal Tyr; peptidomimetics containing a β-residue: DS-70, **2**, and **3**; (**B**) peptidomimetics **Ds13g** (partial agonist) and **Ds13d** (pure antagonist).

1. *N*-acylphenylalanine derivatives (Figure 3A), such as the prototype compound RO0505376. This class comprises the already mentioned Firategrast, an antagonist of α4β1 and α4β7 integrins, designed to reduce trafficking of lymphocytes into the CNS.

The first orally available antagonist of α4 integrin, carotegrast-methyl (Figure 3A), was launched in Japan in May 2022. It exerts an anti-inflammatory effect in vivo by inhibiting both α4β1 and α4β7 integrins expressed on lymphocytes. The efficacy and safety of carotegrast methyl were confirmed in patients with moderate active ulcerative colitis [30].

2. Peptide ligands that reproduce the integrin-binding sequences. FN binds α4β1 integrin through the tripeptide sequence Leu-Asp-Val (LDV), which resides in the alternatively spliced connecting segment 1 (CS-1) region, while the integrin-binding sequence in VCAM-1 is the tripeptide Ile-Asp-Ser (IDS).

The LDVP peptide BIO1211 (Figure 3) [31] demonstrated a potent antagonist effect against α4β1 integrin. It effectively suppressed antigen-induced airway hyper-responsiveness in allergic sheep [32]. The peptide’s affinity is attributed to the α4-targeting diphenylurea pharmacophore MPUPA. However, the stability of BIO1211 is rather poor, as tested in plasma, heparinized blood, and in homogenates of rat liver, lungs, and intestines [33], and is subjected to rapid clearance in vivo [34].

3. Peptidomimetics inspired from the IDS or LDV binding sequences. The stability of the peptide sequence can be improved by adopting a peptidomimetic strategy [35]. Selected examples are depicted in Figure 3. For instance, constraining the essential binding sequences found in VCAM-1 (IDS) and fibronectin (LDV) has afforded a variety of potent cyclic VLA-4 antagonists, such as the Tyr-cyclopeptide **1** (Figure 3) [36].

To cite another widely utilized approach, the introduction of β-amino acids allowed an increase in the enzymatic stability of peptide integrin ligands [37,38,39]. The minimalist MPUPA-β-Pro-Gly DS-70 (Figure 3) was found to be a potent α4 integrin antagonist with noteworthy stability in mouse serum and significant efficacy in an animal model of allergic conjunctivitis [40]. The sequence **2** containing an (*R*)-isoAsp β^3^-core showed low nanomolar affinity for the receptor, being able to prevent the adhesion of FN to Jurkat E6.1 cells, and to prevent FN-induced α4β1 integrin-dependent activation of ERK1/2, AKT, and JNK [41]. Compound **3**, a retro-analogue of BIO1211 containing a dehydro-β-proline ring, was an enzymatically stable, potent inhibitor of α4β1/VCAM interaction, with IC_50_ in the nanomolar range [42]. Figure 3 also reports the commercially available peptidomimetic **Ds13g** and the correlated **Ds13d** (Figure 3B) [43], whose features as integrin ligands are discussed in the next section.

Other orthosteric peptidomimetic α4β1 antagonists have garnered interest for the treatment of dry eye disease [44] and dry age-related macular degeneration (AMD) [45]. For a comprehensive review of the diverse ligands of α4β1 integrin, together with the affinity values reported by the specific biological assay, see Ref. [23].

### α4β1 Integrin Ligands in Diagnostics and Biomaterials

Besides to the therapeutic applications, the utilization of selective integrin ligands can be also harnessed for diagnostic purposes [46] and for biofunctionalization of diverse materials [47]. For instance, the radionuclide peptidomimetic ^64^Cu-CB-TE1A1P-PEG4-LLP2A (^64^Cu-LLP2A) targeting VLA-4 was proposed as a positron emission tomography (PET) imaging biomarker of vaso-occlusive episodes (VOEs) [48].

**Figure 4:**
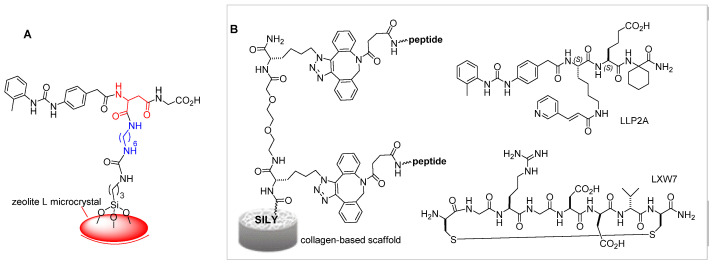
(**A**) Fluorescent zeolite L microcrystal biofunctionalized with an integrin ligand derived from **2**, utilized for coating a leukocyte-responsive nanostructured surface. (**B**) Collagen-based scaffold functionalized with integrin ligand peptides LLP2A or LXW7; SILY (RRANAALKAGELYKSILY-NH_2_) is a high-affinity collagen-binding peptide.

Selective α4 integrin peptidomimetic ligands have been grafted onto monolayers of fluorescent nanoparticles and yielded cell-adhesive devices for detection and quantification of leucocytes expressing active integrins, which can be utilized for monitoring the severity and evolution of diseases such as asthma [49]. A nanostructured surface coated with a α/β hybrid peptide derived from compound **2** (Figure 4A) was able to replicate the high-density multivalency binding between integrin clusters and VCAM-1, displaying notable selectivity for α4β1 integrin-expressing Jurkat cells [50].

A biomaterial was prepared by directly coating expanded polytetrafluoroethylene (ePTFE) with fibronectin-derived LDV peptide [51]. For its flexibility, biostability, and non-adhesiveness, this polymer is widely utilized in clinical applications, such as in the manufacture of blood-contacting implantable devices.

The ligands LLP2A and LXW7 of integrins α4β1 and αvβ3, respectively, have been utilized to modify collagen-based materials. The peptide-modified biomaterial (Figure 4B) improved the adhesion of mesenchymal stem cells (MSCs), osteoblasts, human endothelial progenitor cells (EPCs), and endothelial cells (ECs). In an adult rat model of calvarial bone defect, the peptide-modified biomaterial increased bone formation and vascularization by synergistically regulating endogenous cells with osteogenic and angiogenic potentials. In a fetal sheep model of spinal bone defect, the LLP2A/LXW7-biomaterial promoted bone formation and vascularization, without adverse effects [52].

## 4. Small-Molecule Agonists of α4β1 Integrin

**Figure 5:**
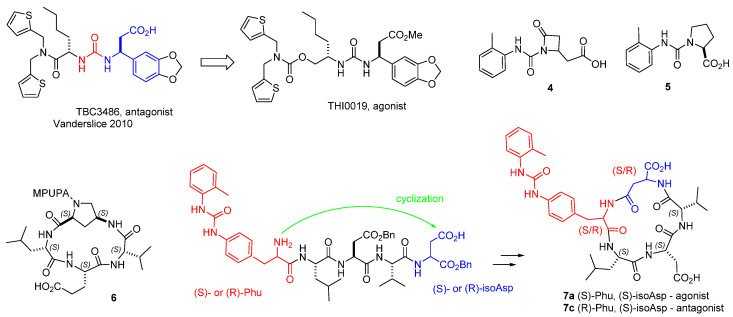
Structures of urea-based integrin agonists: THI0019, **4** and **5**; c[Amp(MPUPA)LEV] (**6**); LDV cyclopeptides designed as mimetics of BIO1211, containing a phenylalanine-urea (Phu) residue (in red), and isoAsp (in blue), (**7**).

Very few potent and selective α4β1 integrin agonists are available at present (Figure 5) [53]. The urea THI0019 was obtained via two structural modifications of the selective antagonist TBC3486 (Figure 5). THI0019 was found to be an agonist, being able to promote cell retention and engraftment [54]. The small ureas **4** and **5** are α4β1 integrin ligands which showed agonistic behaviour [55,56]. Recently, the cyclopeptide 6 was designed by connecting the LDV sequence to a 4-amino-L-proline (Amp) scaffold equipped with MPUPA moiety. Unexpectedly, this compound was able to increase the adhesion of α4β1 integrin-expressing cells (Figure 5) [57].

Very recently, Anselmi et al. conceived a minilibrary of stereoisomeric cyclopentapeptides **7** inspired by the structure of BIO1211 (Figure 5). The α4-targeting diphenylurea moiety was mounted onto (*S*)- or (*R*)-phenylalanine, giving a phenylalanine–urea residue (Phu). The β-amino acid (S)- or (R)-isoaspartate (isoAsp) were introduced aside the LDV peptide to permit cyclization while conserving a carboxylic group, as at the C-terminus of BIO1211. Receptor affinity and selectivity was ascertained by binding assay on purified integrins, while agonism or antagonism was determined by cell adhesion experiments and by checking intracellular signaling (phosphorylation of ERK1/2 in Jurkat E6.1 cells). Intriguingly, while c[(*R*)-Phu-LDV-isoAsp] (**7c**) was a moderate antagonist of both α4 integrins, the all-S configured diasteroisomer c[(*S*)-Phu-LDV-isoAsp] (**7a**) was a potent dual agonist of both α4β1 and α4β7 integrin, whereas c[(*R*)-Phu-LAV-isoAsp] acted as a selective agonist of α4β1 but not of α4β7 integrin, with nanomolar potency [58].

Concerning other leukocyte integrins, small-molecule agonists of integrin αMβ2 were described by Faridi et al. [59,60,61], while the first small-molecule agonist of integrin αLβ2 was discovered by Yang et al. [62].

## 5. Therapeutic Opportunities of α4β1 Integrin Agonists

Agonists of α4β1 integrin could present alternative therapeutic opportunities. The activation of α4β1 integrin might represent an innovative strategy to disturb the migration of leukocytes. After adhesion, leukocyte detachment is required to consent their rolling on the endothelial surface. In this perspective, agonists can preclude the detachment of adherent cells by improving the interaction with integrins [63].

Activation of the integrins on progenitor cells may be a feasible approach to increase the efficacy of stem cell-based therapies by strengthening cell adhesion and engraftment. Agonists can be useful in the treatment of osteoporosis and for the promotion of bone growth due to their specificity for the α4β1 integrin on mesenchymal stem cells and for bone surfaces [64].

The activation of α4β1 integrin could represent a strategy against other diseases. The small-molecule α4β1 integrin agonist THI0019 favoured cell retention and engraftment in stem cell-based therapies [54]. α4β1 Integrin was presumed to play a tumor-protective role in an animal model of colon adenocarcinoma. Indeed, α4β1 depletion led to rapid tumor growth, pointing at small-molecule agonists to influence α4β1 expression level in cancer [65]. Furthermore, both α4β1 and αLβ2 seem to be involved in the localization of cancer-specific CD8+ effector T cells to the local environment of the tumor. Thus, activation of both α4β1 and αLβ2 integrin by means of the small-molecule agonist 7HP349 augmented the accumulation of anticancer T cells to the tumor, improving their efficacy. This effect was boosted by concomitant administration of an anti-cytotoxic T-lymphocyte-associated protein 4 (CTLA-4) agent [66]. The same small-molecule agonist of α4β1 and αLβ2 integrins reported above was tested in combination of a DNA vaccine in a model of Chagas disease. The agonist enhanced both preventive and therapeutic vaccine efficacy, suggesting the adoption of integrin agonists to boost immune response mediated by T cells to different types of vaccines [67].

Pertaining to the other leukocytic integrins, recent evidence supports that αM (CD11b) integrin is deeply involved in the modulation of proinflammatory signaling. Accordingly, αM allosteric modulators stimulating the anti-inflammatory activities of αM integrin can be exploited in the cure of lupus nephritis, a serious illness of systemic lupus erythematosus, marked by penetration of leukocytes to the kidneys [68]. Lastly, the activation of αMβ2 integrin can reduce hypertrophy and mineralization of chondrocytes; therefore, agonists of αMβ2 integrin have been proposed for the therapy of osteoarthritis since they could lead to reduced inflammatory response [69].

The agonist **4** discussed above (Figure 5) was utilized for coating biomaterials to stimulate cell adhesion and promoting tissue repair. Merlo et al. analyzed the effect of the agonist **4** on cell adhesion of equine adipose tissue and mesenchymal stem cells derived from Wharton’s jelly, and studied their adhesion to the agonist incorporated in a poly L-lactic acid (PLLA) matrix [70]. The same agonist was combined with poly(l-lactic acid) (PLLA) nanofibers to consent a controlled release of this molecule, resulting in a medication especially appropriate for skin wounds [71].

## 6. Ligand-Integrin Interactions and Conformational Implications

As anticipated, investigation of α4β1 agonists could provide information about the structural requirements for receptor activation. Up to now, there are over 100 solved structures concerning various heterodimers or α- or β-domains, alone or in complex with different ligands. The large majority of structural analyses performed by X-ray, cryogenic electron microscopy (cryo-EM), NMR, and computations, have been conducted for αvβ3 and αIIβ3 integrins, and contributed to the comprehension of their activation and function. Hence, in the absence of any data for the α4β1 integrin, some deductions can be extrapolated by comparison with RGD-binding integrins, and by analysing the subunits α4 e β1 taken separately from α4β7 and α5β1 integrins.

In general, the α and β subunits consist of a large extracellular ectodomain, a single-span helical transmembrane (TM) domain, and a generally short cytosolic tail. The four domains of the α-chain ectodomain and the seven domains of the β-chain are shown in Figure 1.

In many integrins, including α4β1, the ligand-binding pocket is in a groove at the interface between α and β subunits. A metal-ion-dependent adhesion-site (MIDAS), is situated in the β-subunit. For other integrins, including the β2, MIDAS resides in an extra αI domain of the α-subunit. In RGD-binding integrins, the ligand-binding site comprises a pocket to host Arg located in the propeller domain, and a pocket for Asp carboxylate containing the MIDAS, normally occupied by pro-adhesive Mg^2+^ ion in the βA domain. There are also two regulatory metal binding sites, a ligand-associated metal binding site (LIMBS or SyMBS), and an adjacent to MIDAS (ADMIDAS), each normally occupied by a Ca^2+^ ion (Figure 1C).

In the following sections, structural features of the well-studied integrins αIIbβ3 and αvβ3 are discussed and compared with integrins α5β1 and α4β7. What stands out is that the α4 and β1 subunits show marked differences and specificities as compared with other heterodimers.

### 6.1. αIIbβ3 Integrin

For integrin αIIbβ3, there are almost forty entries in the Protein Data Bank. Despite being the most investigated, there is still some debate about the precise full structure and orientation of the ectodomain relative to the plasma membrane [72,73], due to the adoption of diverse experimental procedures, e.g., the use of detergents or averaging over heterogeneous conformational states, etc.

By default, integrins on the cell surface are in their resting state, so platelets are free to circulate in blood vessels. In the absence of ligands, this state predominates on cell surfaces (>98%) [74]. Acting through their receptors, physiologic agonists stimulate the intracellular binding of talin and kindlin to the cytoplasmic tail of the β-subunit, producing structural rearrangements in the transmembrane (TM) domains that trigger a conformational switch of the ectodomain, generating the active state, competent for extracellular physiologic ligands (“inside-out” activation). Initiation of signals occurs when these ligands bind, and these signals are then transduced through the transmembrane (TM) domains to the cytoplasmic tails. This process regulates cell adhesion and various other functions (“outside-in” signaling).

Figure 1 shows the structures of the whole closed/resting structure of αIIbβ3 (8T2V) [8] (Figure 1A), of the open conformation in the presence of peptide LGGAKQRGDV (2VDR) [9] (Figure 1B), and the carboxylate binding site in the β-subunit, featuring the metal ion dependent adhesion sites (2VDR) [9] (Figure 1C). Details of the ligand binding sites for the complexes αIIbβ3/UR-2922 and αIIbβ3/tirofiban are discussed in the next section.

Springer et al. observed eight distinct RGD-bound conformations of the αIIbβ3 integrin headpiece, by soaking crystals with diverse concentrations of RGD peptides and divalent cations: the closed βI domain conformation, six intermediate βI conformations, and finally the fully open βI with upright hybrid domain. During the extension, diverse regions of the βI domain experienced significant movements, starting with the β1-α1 backbone that hydrogen bonds to the Asp side chain of RGD, followed by ADMIDAS Ca^2+^, α1 helix, α1′ helix, β6-α7 loop, α7 helix, and hybrid domain [75]. In essence, ligand binding at the MIDAS in the βI domain produces a conformational reorganization, transmitted to the opposite end of the βI domain by α7 helix pistoning at the C-terminal connection to the hybrid domain, resulting in its extension (see blue arrows in Figure 1B).

Using cryo-electron microscopy, Hainein et al. determined 3D structures of full-length human integrin αIIbβ3 embedded in a lipid bilayer of nanodiscs, while bound to domains of the cytosolic regulator talin and to extracellular ligands. The authors observed four main conformational states in equilibrium, ranging from compact/bent to two partially extended intermediate conformers and finally to a fully extended state. The results indicate that extension of the ectodomain is possible without separating the legs or extending the hybrid domain, and that the ligand-binding pocket is not occluded in any conformations [76].

In the most recent, full-length structure obtained by cryo-EM in native cell-membrane nanoparticles (Figure 1), surprising features were a fully accessible ligand-binding site and the remarkable distance of the αIIb TM domain from that of β3. Another finding was that the FDA-approved anti-trombotic αIIbβ3 inhibitor eptifibatide, prescribed for myocardial infarction and acute coronary syndromes, induces pseudo-agonist, large conformational rearrangement in the full-length αIIbβ3, plausibly accounting for impaired hemostasis [8].

These studies allow the depiction of a detailed model of the activation process. In ligand-free conditions, Ca^2+^ at ADMIDAS stabilizes the position of the α1 helix at the N-terminus and of the α7 helix at the C-terminus of the βA domain in the closed conformation. Upon inside-out activation, the Ca^2+^-mediated connection between the α1 and α7 helices is broken, allowing an RGD ligand to access to the binding site. Thereafter, the carboxylate of the RGD peptide makes a salt bridge with Mg^2+^ in MIDAS by displacing a water molecule from the coordination sphere of the cation, and by introducing the Arg side chain into the propeller.

Binding the ligand’s carboxylate to MIDAS seems to be fundamental to integrin activation, while pulling by the α subunit may be not mandatory [77]. During receptor opening, the α1 helix-ADMIDAS portion undergoes a large movement (3.9 Å) toward MIDAS, provoking the reorganization of the adjacent loops and the lowering of the α7 helix, pushing the hybrid domain positioned beneath (Figure 1B). The swap of Ca^2+^ with Mn^2+^ at ADMIDAS also separates helices α1 and α7, enabling the switch of βA into the ligand-competent state, but the integrin does not attain the extended conformation.

### 6.2. αvβ3 Integrin

The first crystal structure of the ectodomain of an αI-absent heterodimer in complex with a ligand was reported in 2002 for αvβ3/cilengitide, c[RGDfMeV] (Ca^2+^) [78]. So far, almost twenty structures related to diverse domains of αvβ3 have been deposited in Protein Data Bank.

**Figure 6:**
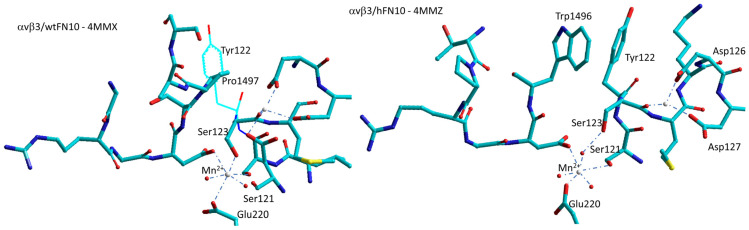
Simplified sketch of the binding site of wild-type FN/αvβ3 (4MMX) and of the binding site of αvβ3 integrin hosting the 10th type III domain of mutant FN (4MMZ), showing the stacking between Trp1496 of FN and Tyr122 of the b-subunit which seems to prevent the dislocation of β1-α1-loop and subsequent receptor extension. Red spheres represent water molecules, grey spheres represent divalent cations.

Arnaout et al. described the first crystal structure of αvβ3 bound to a physiologic ligand, the 10th type III RGD domain of wild-type FN (wtFN10, 4MMX), or to a high-affinity mutant, the pure antagonist (hFN10, 4MMZ). Comparison of these structures revealed a central π–π interaction, between Trp1496 in the RGD-containing loop of the antagonist hFN10 and Tyr122 (β3) of the receptor, that blocks conformational changes and traps the integrin in an inactive conformation (Figure 6).

In the complex with wild-type FN (4MMX), Pro1497 is the closest residue to Tyr122, but in this case, the distance is significantly larger. Removing the Trp1496 or Tyr122 side chains or reorienting Trp1496 away from Tyr122 converted hFN10 into a partial agonist [79]. For hFN10, the hydroxyl oxygen of Tyr1446 of FN also coordinated the Mn^2+^ at ADMIDAS through a water molecule (Figure 6).

Apparently, the π–π interaction between Trp1496 and Tyr122 was crucial for blocking conformational changes induced by the binding of ligand, as shown in mutational studies. This hypothesis was substantiated by subsequent work, aimed at converting the partial αvβ3 agonist MK-429 into the pure antagonist TDI-4161. The structure of MK-429 was modified by introducing a large bicyclic benzo[d]thiazole capable to inducing a π–π stacking with Tyr122 [80].

### 6.3. α4β7 Integrin

As said for α4β1, also α4β7 integrin is involved in leukocytes rolling and adhesion, as well as in its firm adhesion on blood vessels. α4β7 Integrin mediates rolling adhesion in Ca^2+^, while in Mg^2+^ or Mn^2+^, leukocytes adhesion becomes firm.

Springer et al. have reported the structures of the headpiece of α4β7 with the antibody natalizumab (4IRZ), or with the antibody Fab ACT-1 (3V4P), or with the small-molecule RO0505376 and Fab ACT-1 (3V4V) [81]. The inspection of the crystal structure of natalizumab (3V4P) with α4β7 revealed that natalizumab binds to the β-propeller of the α4 subunit in a position which is close but alternative to the orthosteric ligand-binding site. In contrast, the ACT-1 antibody binds to the β7 subunit in the proximity the ligand-binding site (3V4P), thus explaining why natalizumab inhibits both α4β1 and α4β7, while ACT-1 only inhibits α4β7.

**Figure 7:**
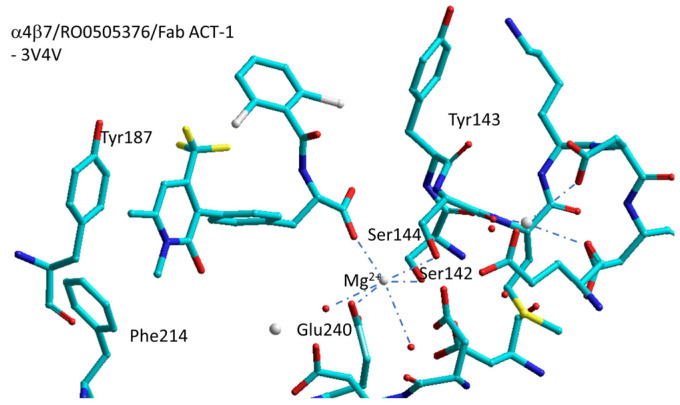
Sketch of the binding site of RO0505376 in α4β7integrin/Fab ACT-1 (3V4V). Red spheres represent water molecules, grey spheres represent divalent cations.

RO0505376 is an antagonist of α4β1 and α4β7 integrin with IC_50_ values of 32 nM and 42 nM, respectively. The ternary complex formed by α4β7/RO0505376/Fab ACT-1 is contained in 3V4V (Figure 7) [82]. The shape of the binding cleft is clearly dissimilar from that of the RGD-binding integrins. The carboxylate of RO0505376 interacts with the MIDAS cation forming two NH-backbone hydrogen bonds (Tyr143 and Asn235 of β7). The amide NH of the inhibitor forms an H-bond with backbone carbonyl of Asn235 with an orthogonal 2,6-dichlorophenyl moiety in the proximity of the specificity determining loop. The pyridone ring forms π–π stacking with Phe214 and Tyr187 of α4 and an H-bond with Ser238 of β7.

### 6.4. α5β1 Integrin

Compared with the other RGD-binding integrins, the β1 transmit quite different types of signals into cells. α4β1 Integrin and RGD-binding αVβ1 and α5β1 require markedly different tension thresholds to support cell spreading [83]. Six X-ray crystal structures related to α5β1 in PDB have been reported so far. The inspection of these structures underlines some structural distinctive features worthy of notice. The comparison between the β1 and β3 subunits shows that the former shows an expanded binding pocket, because the residues Arg214 and Arg216 in the β3 subunit are replaced with Gly217 and Leu219 in β1. Unlike the resting structures of β3 integrins, α5β1 integrin exhibits a half-bent conformation [84,85]. The integrin headpiece of α5β1 is more stable in the closed state than the ectodomain and is thus more difficult to activate [86].

In 2012, Nagae et al. reported two structures of the α5β1 headpiece bound to the allosteric inhibitory antibody SG/19 Fab, i.e., a ligand-free form (3VI3), and an RGD complex (3VI4) [87]. The latter was characterized by a conformational state intermediate between closed and open.

**Figure 8:**
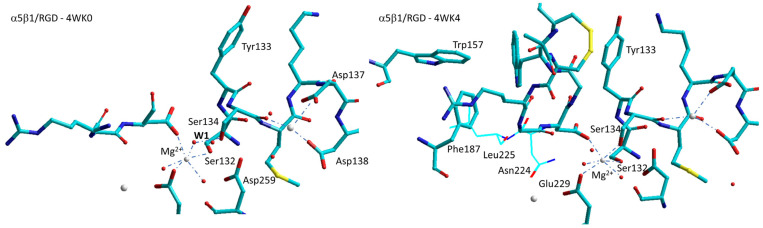
Sketches of the metal-ion-dependent adhesion sites for the complexes α5β1 with a linear RGD peptide (4WK0), and with an RGD cyclopeptide (4WK4). The latter differs particularly in the repositioning of Ser134 to displace water1 (W1) at MIDAS. Red spheres represent water molecules, grey spheres represent divalent cations.

Subsequently, Springer at al. performed soaking experiments and obtained structures with linear or cyclic RGD peptides (4WK0, 4WK2, 4WK4, plus 4WJK with no peptide) [88]. While the linear RGD peptide induced no movement in the β1-α1 loop (4WK0, Figure 8), in the absence of added Ca^2+^ the same ligand produced a measurable movement of the β1-α1 loop and α1-helix, while Ser134 displaced a water molecule at the MIDAS (4WK2). On the other hand, binding of a disulfide-cyclized RGD peptide with 20-fold higher affinity in Ca^2+^ produced a noteworthy conformational change in the β1-subunit βI domain, as well as the movement of the Ser134 side chain into direct coordination with the MIDAS, a conformation that is intermediate between low-affinity/closed and high-affinity/open (4WK4, Figure 8).

These results support the role of ADMIDAS as a negative regulatory site responsible for integrin inhibition by high concentration of Ca^2+^ and for activation by Mn^2+^. It has been proposed that during the process of extension/activation of β1 integrins, Ca^2+^ at the ADMIDAS site becomes highly instable, until it is removed from the site, whereas the cations of LIMBS and MIDAS maintain their positions [88].

On resuming, ADMIDAS seems to be an important factor in the transmission of conformational changes between the βI and hybrid domains. While the MIDAS Mg^2+^ ion binds the Asp side chain of the RGD motif, the nearby Ca^2+^ ions in the ADMIDAS and SyMBS regulate ligand affinity. Studies with diverse integrins confirmed that low concentrations of Ca^2+^ (∼50 μM) enhance binding and higher concentrations of Ca^2+^ (in the range 1–10 mM) inhibit binding. Replacement of the cations with Mn^2+^ has the net effect of substantially enhancing integrin binding.

The roles of Mn^2+^ and ADMIDAS in regulating integrin affinity in integrin α5β1 have been studied using antibody Fab fragments specific for the open conformation. As it turned out, Mn^2+^ seems to markedly increases both the population of the extended stateand the intrinsic affinity of the α5β1 extended state for the ligand. However, the extended state is populated only partially. That is, Mn^2+^ modifies the conformational ensembles only in part. The study also confirmed the role of ADMIDAS in enlarging the difference in affinity of the low-affinity, of the extended-closed, and the high-affinity extended-open states [89].

### 6.5. Agonism or Antagonism and Dynamics Nature of MIDAS

Very recently, Springer et al. reconsidered the role of the water molecules present in MIDAS as discussed above and proposed a simple but robust theory to explain why small-molecule inhibitors previously regarded as potential therapeutic entities failed in late-stage clinical trials for chronic indications. Plausibly, this may be caused by partial agonism at low concentration, namely the stabilization of the high-affinity, extended-open integrin conformation. The authors utilized diverse techniques, including size-exclusion chromatography in the absence or in the presence of ligands, to measure any increase of the hydrodynamic radius, relatable to extension of the whole structure [17]. In addition, the effects of the ligands on the conformation of the receptor were examined by means of MBC319.4 antibody, specific for the extended states of β3 integrins.

**Figure 9:**
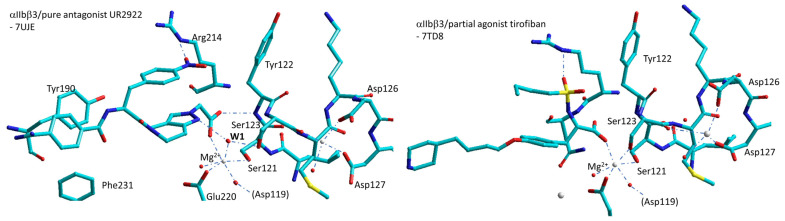
Simplified sketches of the MIDAS and ADMIDAS region of complexes αIIbβ3/UR-2922 and αIIbβ3/tirofiban. The “closing” antagonist UR-2922 stabilizes water1 between the MIDAS metal ion, the nitrogen N2 of the 1H-pyrazole ring, and Ser123 sidechain. With the opening ligand tirofiban, β1-α1 loop movement brings Ser123 to displaced water1 (W1) to coordinate directly to the MIDAS. Red spheres represent water molecules, grey spheres represent divalent cations.

As a result, failed small-molecule inhibitors of integrins αIIbβ3 appear to stabilize the open/high-affinity conformation. The reasons of this behaviour have been investigated by analyzing the crystal structures of the αIIbβ3 headpiece in complex with a variety of ligands, such as the “opening” ligands Roxifiban, Lotrafiban, Tirofiban, and many others, in Mg^2+^ or Mn^2+^, in comparison with “closing” ligands such as UR-2922, Gantofiban, etc. During headpiece extension, the key early step is the movement of the β1-α1 loop toward the αIIb subunit. In the absence of a ligand, the sidechain oxygen of Ser123 in the MIDAS motif hydrogen is coordinated to water1. In the subsequent open states, movement of the β1-α1 loop repositions the Ser123 sidechain oxygen to take the place of water1 (Figure 9). Apparently, the pure closing inhibitors might be longer and more rigid than RGD and block this movement.

An extensive analysis of the crystal structures of the αIIbβ3 headpiece in complex with inhibitors that either stabilized integrin headpiece opening or closure, or were conformationally neutral (in Mg^2+^ or Mn^2+^), supports a simple structural feature present in diverse αIIbβ3 antagonists that stabilizes integrins in their bent-closed conformation. Closing inhibitors contain a polar nitrogen that stabilizes water1. Other “closing” antagonists such as BMS4 indirectly stabilize water1 through water2 by means of the nitrogen N1.

In contrast, with opening ligands, including tirofiban (Figure 9), eptifibatide, lotrafiban, and EF-5154 in Mg^2+^ and roxifiban in Mn^2+^, the movement of the β1-α1 loop brought the Tyr122 backbone into position to hydrogen bond to the drug carboxyl group, while Ser123 displaced water1 to coordinate directly to the MIDAS metal ion (Figure 9).

It was proposed that expulsion of this water1 molecule is a requisite for transition to the open conformation also for α4 integrins. The experiments were carried out for the dual α4β1/α4β7 integrin antagonist firategrast, the commercially available peptidomimetics **DS13g** and the correlated **Ds13d** (for the chemical sketches, see Figure 3) [43]. With respect to the piperidine ring in **Ds13d**, **Ds13g** contains an extra basic nitrogen in the piperazine ring. While firategrast and **Ds13d** stabilize the open integrin conformation, **DS13g** stabilizes the closed conformation.

## 7. Simulations with Homology or Composite Models of α4β1 Integrin

As anticipated in the previous paragraph, the detailed structure of α4β1 integrin is not yet accessible. An alternative to conducting pharmacodynamics investigations is to perform molecular docking simulations. To that end, either homology or composite models can be built. Initial attempts by the homology modelling approach required extensive optimization steps.

Dixon et al. obtained a homology model of the β1 subunit based on the I domain of the integrin CD11B subunit and analyzed the binding of the LDV cyclopeptide and compound TBC3486 (Figure 5) [90]. Macchiarulo et al. simulated the binding of the *N*-acylphenylalanine derivative TR-14035 using a model based on the structure of αvβ3. The so-obtained structure closely reproduced the binding of RO0505376 in α4β7 (Figure 7) [91]. More recently, the deep-learning method AlphaFold was used to generate an optimized homology model of the integrin [92].

Frequently, simulations with α4β1 models have been conducted for in silico screening to select potential inhibitors of the same integrin [93,94] or other β1 integrins [95]. For instance, molecular docking was used to assist in the design of a blocking polypeptide (antifibrotic 38-amino-acid polypeptide, AF38Pep) for specific inhibition of extra domain A-FN associations with the fibroblast-expressed α4 integrins [96].

The availability of the crystallographic structures of α4β7 and α5β1 integrins allowed for obtaining the structure of the heterodimer by merging the α4 and β1 subunits. To create the chimeric receptor, the crystal structure of the α4β7 headpiece in complex with Fab ACT-1 and RO0505376 (3V4V) [82] is generally utilized. For α5β1, the situation is more complicated. There are diverse high-resolution crystal structures available of headpiece domains with an RGD peptide (see above). The binding modes of the RGD peptides with respect to the β1-α1 loop are somewhat different, since they refer to closed/inactive or partially open conformations. In two out of four structures (4WK2 and 4WK4) [88], Mg^2+^-bound water is replaced by Ser134.

In addition, it must be stressed that the assembly of the separate subunits requires a precise optimization of the contacting regions. Finally, simulations of integrins remain particularly challenging because the typical molecular mechanics force fields employed to simulate ligand−receptor interactions fail in the calculation of the metal coordination sphere.

In summary, the receptor models as reported in the literature for performing molecular docking greatly vary, depending on the homology modelling or chimeric approach, of the specific β1 crystal structure selected, the level of heterodimer optimization, and the computational method, which can range from specialized molecular mechanics, to ab initio quantum mechanics, to hybrid methods [92]. For these reasons, it is not completely unexpected that structurally correlated compounds, such as, for instance, derivatives of the LDV peptide BIO1211, yielded rather diverse alternative receptor-bound structures, as reviewed in the following paragraphs.

In a very recent study, Baiula et al. analyzed by molecular docking a minilibrary of minimalist analogues of BIO1211, in which the MPUPA-LDVP sequence was reduced to MPUPA-β-residue-Gly, including the previously mentioned MPUPA-β-Pro-Gly (DS-70) (Figure 3) [41]. This strategy generated several pure antagonists with IC_50_ affinity values in a low nanomolar range, such as MPUPA-(*R*)-isoAsp(*N*-propyl)-Gly (**2**, Figure 3). These compounds reduced the adhesion of integrin-expressing cells to the natural ligands, without inducing receptor open conformation, nor activation of intracellular signaling pathways.

**Figure 10:**
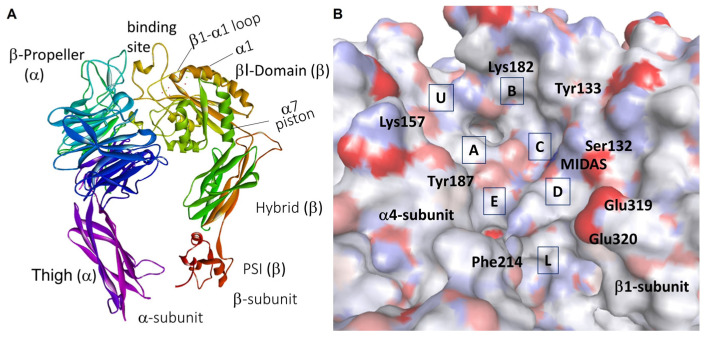
(**A**) Ribbon image of the chimeric α4β1 model from Ref. [41], and (**B**) details of the binding site rendered by the solvent accessible surface, showing subpockets A–E, U, L; the positions of relevant residues are also shown.

To obtain the inactive structure of the receptor, the authors paired by protein–protein docking the α4 and β1 monomers taken from PDB ID 3V4V and PDB ID 4WK0, respectively. In the obtained model (Figure 10A), the putative ligand binding site resides in a vertical crevice at the α/β interface, as shown for α4β7 integrin. This groove can be partitioned into subpockets of different size, shape, and composition of delimiting residues (Figure 10B): the middle subpockets A–E, plus lower (L) and upper (U) subpockets [41,97].

**Figure 11:**
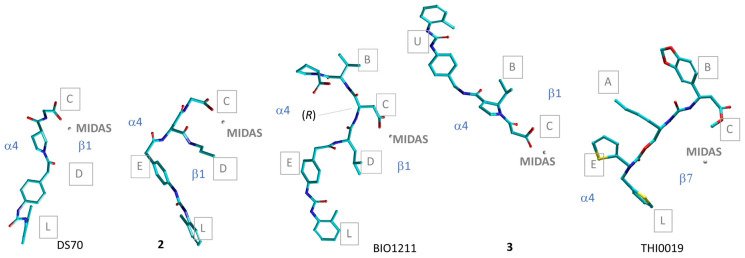
Comparison of the predicted binding poses obtained by molecular docking for the MPUPA antagonists DS-70 and **2** [41], **3**, [57], BIO1211 as reported in Ref. [94], and the agonist THI001 [54]. A–E, U, L refer to binding sites subpockets as shown in Figure 10.

In general, in the predicted poses, the ligands lie vertically along the α4β1 crevice, with the ligand’s carboxylate well inserted into subpocket C, so that the main interaction is the salt bridge with Mg^2+^. As prototypical examples, the sketches of the bioactive conformations of DS-70 and **2** are shown in Figure 11. For both, the diphenylurea group finds a place in the lower subsite L of the receptor, while the peptide portions attain interactions with the β1 subunit. For the minimalist peptide **2**, the propyl substituent is allocated in subpocket D.

The bioactive conformation of **2** can be compared to that obtained by da-Silva et al. for MPUPA-LDVP (BIO1211, Figure 3) using a homology model of α4β1 integrin [94]. In the calculated pose, the ligand’s backbone folds in a reverse S-shape, and AspCOO^−^ of the peptide makes a salt bridge with Mg^2+^ at MIDAS. The C-terminal Pro occupies a space between subsites B and U on the top of the cavity, while the N-terminal MPUPA finds its position into subpockets E and L (Figure 11). Unfortunately, this structure has the fatal flaw that Asp residue appears in the (*R*) absolute configuration.

An alternative display of MPUPA-peptides was calculated by Sartori et al. [57]. Using the α5β1 integrin complex (3VI4) [87] as a template, the α4 subunit extrapolated from α4β7 (3V4V) was aligned with α5, and the model was exploited for docking eight known α4β1 integrin antagonists, including BIO1211 and **3** (for the chemical structures, see Figure 3). For these compounds, the MPUPA group occupied the upper subsite U. Figure 11 reports the situation for ligand **3**. This procedure was used to pre-screen new potential ligands, and led to the unexpected identification of the agonist c[Amp(MPUPA)LEV] (**6**, Figure 5).

### Simulations of α4β1 Integrin Agonists

Limited research has been devoted to agonists of leukocyte integrins. The binding poses of small αMβ2 agonists composed of a 3-benzylthiazolidine-2,4-dione scaffold connected to a 4-(furan-2-yl)benzoic acid were studied by molecular docking. According to the simulations, the ligands were projected to identify a hydrophobic cleft near the ligand-binding site, indicating the possibility of an allosteric mechanism. [59].

Molecular docking analysis was also performed for the agonist urea THI0019 (Figure 5), IC_50_ in the 1–2 mM range, capable of enhancing the adhesion of α4β1-expressing cell lines to VCAM-1 and the CS-1 region of FN. THI0019 was docked into the available α4β7 crystal structure (3V4V), and the predicted binding site overlaps the classic binding pocket (Figure 11). Thus, the hypothesis is proposed that for the natural ligand to bind, the compound would need to be displaced from this site [54].

A recent work committed particular attention to the structural requisites at the basis of agonism or antagonism at α4β1 integrin for LDV cyclopeptides [58]. Among the cyclopeptides **7** (Figure 3), the agonist **7a**, c[Phu-LDV-isoAsp], and the antagonist **7c**, c[(*R*)-Phu-LDV-isoAsp] (Phu, phenylalanineurea), captured attention for their opposite behaviour, despite the very close similarity. The manner in which the agonist boosts, whereas the antagonist diminishes, both intracellular signaling and the adhesion of α4β1 integrin-expressing cells to the native ligands FN or VCAM-1, appears particularly puzzling.

Molecular modeling of the prototypical agonist **7a** and antagonist **7c**, plus diverse correlated derivatives, was performed on a α4β1 integrin receptor model obtained by combining the α4 subunit from PDB 3V4V with the β1 subunit from PDB 4WK4, which describes a state of α5β1 intermediate between low affinity/closed and high affinity/open [88]. Hybrid density-functional theory (DFT) combined with QM/MM calculations was employed to address the MIDAS region and the receptor residues in the binding site. The poses obtained with Autodock 4.0 were equilibrated by a short molecular dynamics (MD) simulation.

**Figure 12:**
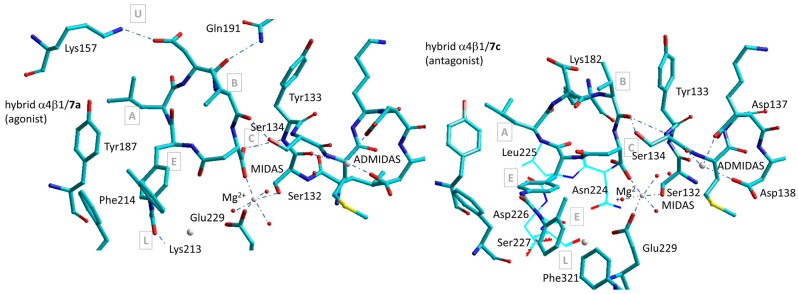
Predicted complexes between composite α4β7/α5β1 receptor and the agonist **7a**, c[Phu-LDV-isoAsp] (**left**), and the antagonist **7c**, c[(*R*)-Phu-LDV-isoAsp] (**right**) [58]. A–E, U, L refer to binding sites subpockets as shown in Figure 10. Red spheres represent water molecules, grey spheres represent divalent cations.

The bioactive conformations of **7a** and **7c** are sketched in Figure 12. The macrolactam rings of the two compounds occupy the same subsites A–C and D as established in Figure 10, while the diphenylurea group find a place in the lower subsite L of the crevice. Unexpectedly, the coordination to Mg^2+^ in the MIDAS involves the carboxylate of isoAsp instead of that of Asp [58].

Indeed, for the all-L configured agonist **7a**, c[Phu-LDV-isoAsp], AspCOO^−^ interacts with Lys157NHζ+ (α4) by a salt bridge. This matches with the experimental finding that the carboxylic group of isoAsp, as opposed to that of Asp, was crucial for maintaining high receptor affinity. Overall, it can be noted that most of the stabilizing interactions of agonist **7a** involve residues of the α subunit (Tyr187, Phe214, Lys213).

In contrast, the antagonist c[(*R*)-Phu-LDV-isoAsp] (**7c**) makes few contacts with the α subunit, compensated by tight interactions with residues of the β subunit (Figure 12). The diastereoisomer **7c** differs from **7a** only for the (*R*)-configuration at Phu. The ionic bond AspCOO^−^-Lys182NHζ+ (β1) seems to clasp the cyclopeptide scaffold to the β1 subunit, plausibly playing a certain role in the antagonist behaviour. Indeed, replacing Asp with Ala resulted in the transformation of the antagonist **7c** into a modest agonist [58]. In the two complexes, the positions and interactions of the residues surrounding MIDAS present distinctive features. To our surprise, the coordination sphere of Mg^2+^ cation includes three water molecules, but not Ser134. In both cases, the hydroxy group of Ser134 is hydrogen-bonded to the ligand’s Val(C=O). In cyclopeptide **7a**, the second Asp carboxyl oxygen is bound to the amide NH of Tyr133, while in **7c**, it forms a pseudo γ-turn with (*R*)-PhuNH, an effect of the reversal of configuration at this residue [58].

The characteristics of complexes shown in Figure 12 can be interpreted in light of the crystallographic data above reported for RGD-binding integrins. In general, it has been established that the mechanism of integrin activation requires a specific reorganization of a pre-existing network of interactions around the β1-α1 loop of the β subunit. In this perspective, antagonism by **7c** might be the result of the complete insertion of a macrolactam ring into the binding pocket, making tight contacts with the MIDAS region and, in particular, Tyr133, resulting in freezing the positions of the βI domains. In this perspective, the transmission of the activation signal by the downward movement of the α7 helix and the outward swing of the relative hybrid domain, as depicted in Figure 1B, cannot take place [98]. Conversely, the reversed absolute configuration at Phu compels **7a** to dock cornered into the binding site, establishing fewer contacts with components of the β1 subunit. Hence, Tyr133, the β1-α1 loop and other elements of the βI domain seem to have room for the dislocation necessary to receptor activation.

Comparison of the two complexes shows a modest approach of Tyr133 and the β1-α1 loop towards the α4 subunit in the α4β1/**7a** complex, as expected for an agonist [75]. On the other hand, the cations at MIDAS and ADMIDAS seems to have a slightly increased distance, approximately 0.8 Å greater compared with the α4β1/**7c** complex. [58]. This is in contrast to the crystallographic evidence for RGD-binding integrins, for which a noteworthy movement of ADMIDAS toward MIDAS has been observed [75]. For β1 integrins, such moderate separation when complexed to agonist **7a** might make sense. Within β1 integrins, the presence of Ca^2+^ in the ADMIDAS is implicated as a negative regulatory site responsible for inhibiting integrin activity. It has been proposed that, during the activation of β1 integrins, Ca^2+^ at the ADMIDAS site becomes highly mobile and is eventually expelled, while the status of Ca^2+^ at LIMBS and MIDAS remains unchanged [88]. In line with this observation, an examination of the solid, water-accessible surface of α4β1/**7a** reveals that the exposure of Ca^2+^ in the ADMIDAS is greater compared with that of α4β1/**7c** [58].

It must be noticed that the simulation of the agonist **7a** did not reveal large reorganizations of βI and hybrid domains compatible with activation. This failure can be ascribed to inherent limitations of the computational approach, in particular, the practical difficulties to perform long MD simulations. Also, there is evidence that in α5β1 integrin, the binding of small peptide ligands is not sufficient for full opening [88]. The extended, open conformation is observed only when both Mn^2+^ and FN are present [99], while Ca^2+^ binding to the ADMIDAS seems to stabilize the closed conformation. Plausibly, the presence in the calculated model of Ca^2+^ at ADMIDAS could contribute to preventing a larger conformational transition.

## 8. Conclusions

Inhibitors of intracellular signaling and cell adhesion mediated by α4β1 integrin are regarded as real or promising tools for the treatment of inflammatory diseases, multiple sclerosis, asthma, allergic conjunctivitis, and dry eye disease. On the other hand, the agonists of α4β1 integrins have attracted attention for their potential to impede the recruitment of circulating leukocytes by consistently inhibiting their rolling onto the endothelial surface, thereby hindering their ability to reach sites of inflammation. Also, the activation of α4β1 seems to be a promising strategy to improve cell retention and engraftment in stem cell-based therapies.

At present, very few effective and selective agonists of the α4 integrins have been reported. As a consequence, very little is known about ligands’ characteristics at the basis of receptor blockade or activation. The situation is particularly unclear as the exact structure of the α4β1 integrin has not been revealed. Extensive crystallographic studies conducted for the RGD-binding integrins αvβ3 and αIIbβ3 led to a quite precise depiction of the activation mechanism. However, the proposed models cannot be simply translated to the non-RGD-binding α4β1 integrin.

In summary, it is generally accepted that small molecules bind to the integrins without inducing the conformational transition necessary for activation. However, there is evidence that at low concentrations, several small ligands regarded as antagonists act as partial agonists and promote active conformation of the integrin. Other small molecules acting as pure antagonists appear to stabilize the inactive conformation by freezing the reorganization of the dynamic interactions about MIDAS and the dislocation of the β1-α1 loop and ADMIDAS. The extended conformation of the receptor is better stabilized by ligands with comparatively larger structures, e.g., peptides or cyclopeptides [88], capable of attaining significant interactions with the receptor with high affinity.

Remarkably, the recently reported family of cyclopeptides **7** yielded some potent agonists, being able to increase both intracellular signaling and the adhesion of α4β1 integrin-expressing cells to the native ligands FN or VCAM-1. These cyclopeptides consented also to investigate the structural features and the 3D display of the pharmacophores at the basis of the interactions with the receptor. Molecular docking on cyclopeptides **7** suggests a depiction of pure antagonism as the result of tight contacts with the β1 subunit that immobilizes Tyr133 and MIDAS, while agonists mainly interact with the α4 subunit, making room for the movement of the β1-α1 loop. Simulations failed to reproduce receptor extension. Nonetheless, these agonists might act as promoters of the interactions between the integrin and FN or VCAM-1 by predisposing the receptor to a semi-activated conformation and by facilitating Ca^2+^ expulsion. The complete reorganization of the integrin structure could occur solely through subsequent binding with FN or VCAM-1.
